# Porcelain Veneers in Vital vs. Non-Vital Teeth: A Retrospective Clinical Evaluation

**DOI:** 10.3390/bioengineering10020168

**Published:** 2023-01-28

**Authors:** Maciej Zarow, Louis Hardan, Katarzyna Szczeklik, Rim Bourgi, Carlos Enrique Cuevas-Suárez, Natalia Jakubowicz, Marco Nicastro, Walter Devoto, Marzena Dominiak, Jolanta Pytko-Polończyk, Wioletta Bereziewicz, Monika Lukomska-Szymanska

**Affiliations:** 1 “NZOZ SPS Dentist” Dental Clinic and Postgraduate Course Centre, 30-033 Cracow, Poland; 2 Department of Restorative Dentistry, School of Dentistry, Saint-Joseph University, Beirut 1107 2180, Lebanon; 3 Department of Integrated Dentistry, Institute of Dentistry, Faculty of Medicine, Jagiellonian University Medical College, Montelupich 4, 31-155 Krakow, Poland; 4 Department of Biomaterials and Bioengineering, INSERM UMR_S 1121, Biomaterials and Bioengineering, 67000 Strasbourg, France; 5 Dental Materials Laboratory, Academic Area of Dentistry, Autonomous University of Hidalgo State, Circuito Ex Hacienda La Concepción S/N, San Agustín Tlaxiaca 42160, Mexico; 6 “Studio Nicastro” Dental Clinic, Corso Trieste 142, 00198 Roma, Italy; 7 Independent Researcher, 16030 Sestri Levante, Italy; 8 Department of Dental Surgery, Silesian Piast Medical University, ul. Krakowska 26, 50-425 Wrocław, Poland; 9 Department of General Dentistry, Medical University of Lodz, 251 Pomorska St., 92-213 Lodz, Poland

**Keywords:** anterior restorations, ceramic, dental veneers, follow-up, non-vital teeth, porcelain laminate veneers, vital teeth

## Abstract

Nowadays, the ceramic veneer approach can be considered more predictable than direct composite veneer. To date, there is a lack of studies comparing the clinical performance of anterior veneers cemented on vital teeth (VT) and non-vital teeth (NVT). This longitudinal clinical study investigated the performance of ceramic veneers in VT or anterior NVT. A total of 55 patients were evaluated in the study. Two groups were defined based on the vitality status of the teeth (93 teeth—vital and 61 teeth—non-vital). The United States Public Health Service (USPHS) criteria were used to assess the clinical status. The data were evaluated statistically with the Mann–Whitney U test. All restorations were considered acceptable, and only one veneer in VT failed for the criteria of secondary caries. There were no statistically significant differences in any of the criteria evaluated (*p* ≤ 0.671). The ceramic veneers evaluated showed a satisfactory clinical performance both in VT and NVT.

## 1. Introduction

Facial and dental aesthetics are currently considered crucial for patients aiming to boost their self-confidence [1,2]. Displeasure with tooth color and shape has amplified the request for aesthetic dental approaches. There are two frequently applied and non-invasive options available to resolve aesthetic problems in contemporary dentistry, namely, direct composites and porcelain veneers [3].

On the one hand, direct composite veneers can be a perfect, minimally invasive, and long-lasting treatment to enhance the color, shape, and incisal embrasures of the teeth [4,5]. The tooth preparation can often be avoided, and direct non-preparation composite veneers can be performed. This solution is especially indicated for adolescent and young patients. Moreover, composite veneers can be easily repaired and corrected if color or shape alterations are needed. Additionally, the hardness and wear resistance of composites are more similar to enamel than porcelain, and accordingly, this material may be preferred to restore mandibular anterior teeth. The anatomical stratification of resin composite along with the application of tints and/or opaquers helps to mimic tooth color, providing an aesthetic appearance [4]. This could be possible in one visit, and the key conditions to attain these cases are to understand dental morphology and to master the diverse layers of resin composite [4]. It should be emphasized that there are some drawbacks associated with the application of resin composite, namely, marginal leakage, low wear resistance, inferior color stability, susceptibility to discoloration, or difficulty in the removal of excess material [6,7,8]. However, composite veneers are an excellent choice in cases of small tooth repairs, including small chips, minor misalignment, slight discoloration, tooth shape correction, and diastema closure [4,8]. As a relative contraindication, severe discoloration can be considered when opaque materials are applied to mask the discolored tooth structure; thus, the final aesthetic outcome can be compromised. Additionally, occlusal risk factors (occlusal dysfunction, constricted chewing pattern, bruxism, parafunctions, etc.) and severely structurally compromised teeth may also be regarded as contraindications [9]. Moreover, the long-term success of direct composites may depend on patient selection, cavity location and size, material choice, and operative technique [5,7,8].

On the other hand, ceramic veneers have become the primary choice for patients when color alteration (i.e., tetracycline discoloration, non-vital tooth), space closure, shape correction, and the reconstruction of worn, misaligned, malformed, or fractured teeth are needed [3,10,11,12]. Nowadays, the ceramic veneer approach can be considered more predictable than direct composite veneers in the case of discolored teeth due to laboratory manufacturing and enhanced ceramic properties. Moreover, comprehensive treatment including numerous teeth and complex smile correction (inclination of the axis of individual teeth, relationship of the central incisors to the lateral incisors, etc.) can be meticulously planned and executed in cooperation with the dental technician to achieve perfect final restorations. These restorations can absolutely imitate the characteristics of the tooth structure [3,13], providing good mechanical properties, high aesthetics, biocompatibility, and long-term clinical performance [14,15,16,17,18,19]. Additionally, minimally invasive preparation and the easy removal of cement excesses along with the ceramic material exhibiting enhanced properties turn this treatment option into a preferred solution. In contrast, it requires more appointments and is more expensive and difficult to repair in the case of ceramic chipping or breakage.

In turn, resin composite veneers can be used as an alternative choice for ceramic veneers in the anterior area; however, limited longevity can affect the final aesthetic outcome of the restoration [20,21]. On the contrary, ceramic veneers (especially felspathic porcelain veneers) offer a durable aesthetic result due to the ability to reproduce the luster of natural teeth and the life-like appearance of the patient [14,22]. The selection of indirect ceramic restoration provides aesthetic reconstructions with higher abrasion resistance, biocompatibility, color stability, appropriate translucency, exceptional marginal integrity, and contour stability. Further, one should state that the preparation is not subgingival in most of the veneer cases, and considering this, porcelain veneers are associated with a low risk of gingival irritation owing to a hindered plaque accumulation on the restoration surface and at the interface [23]. 

To date, there is a lack of studies comparing the clinical performance of anterior veneers cemented on vital teeth (VT) and non-vital teeth (NVT). Therefore, the aim of this retrospective study was to investigate the clinical behavior of indirect porcelain ceramic veneers cemented on vital and non-vital anterior teeth.

## 2. Materials and Methods

### 2.1. Study Characteristics, Participants, and Design

The study was designed as a retrospective evaluation of indirect porcelain veneers cemented on VT and NVT. Informed consent was obtained from all individuals. The recall took place between January 2019 and July 2022. The inclusion criteria were as follows: veneers made from feldspathic porcelain by one dental technician and cemented by one restorative dentist, stable occlusion, full dentition, anterior teeth without occlusal overloading (no sensation of fremitus), VT with confirmed vitality status, NVT with acceptable root canal filling, the absence of periapical lesion, and the presence or lack of fiber post. The exclusion criteria included: subgingival class V restoration beyond cemento–enamel junction, patient under the age of 25 years, and composite restorations exceeding 50% of the adhesive surface. The study obtained the permission of the ethical commission of Jagiellonian University (no. 122.6120.60.2016).

The study population consisted of 55 patients restored with anterior porcelain veneer restorations. Two groups were defined based on the vitality status of the teeth. The VT group consisted of 25 patients (18 females, 7 males) with a mean age of 51.03 years. In total, 93 VT (38 central incisors including only one mandibular incisor, 37 lateral incisors, and 18 canines) were evaluated after a mean observation period of 8.3 years. The NVT group consisted of 30 patients (24 females, 6 males) with a mean age of 46.2 years. In this group, a total of 61 teeth (43 central incisors, 16 lateral incisors, and 2 canines) were evaluated after a mean observation period of 7 years. The distribution according to patient-related factors is shown in Table 1.

### 2.2. Pre-Treatment Procedures

All restorations were changed to new ones according to indications. In all cases, the color was evaluated by both the dental technician and clinician before starting the porcelain veneer preparation. Additionally, photographs of the tooth before and after preparation were obtained.

The shape of the new porcelain veneers was tested by the mock-up procedure. Transferring the shape of the tooth from the wax-up was performed by means of a silicone index I (Zeta Plus L, Zhermack, Badia Polesine, Italy). The silicone excess was cut away with a surgical scalpel or a straight handpiece carbide bur, and then a composite temporization material (Protemp, 3M ESPE, St. Paul, MN, USA) was applied to the index and was introduced on the teeth. After the composite resin had fully set (about 5 min), the silicone index was gently removed and the excess material on the palatal side and the proximal surfaces was discarded. 

Additionally, the silicon index II (Zeta Labor, Zhermack, Italy) was performed based on the diagnostic wax-up and cut with a scalpel no 10 (Swann Morton, Sheffield, England) into two parts in order to control the tooth reduction. 

### 2.3. Tooth Preparation Procedure

The porcelain veneer preparation was made from the temporary mock-up and used additional index II as a control. All of the preparation procedures were performed under local anesthesia (Ubistesin™ Forte Local, 3M ESPE, St. Paul, MN, USA). Horizontal grooves were created on the labial surface and vertical ones on the incisal edge of the mock-up using burs no. 868B018 and 68016 (Komet Brasseler, Lemgo, Germany) on an electric red ring 1:5 increasing contra-angle handpiece with copious water cooling. After the removal of the mock-up under loupe magnification (Zeiss 4.3, Oberkochen, Germany), the minimal invasive outline of the preparation (less than 0.2 mm) was performed using a round ball diamond bur (bur no. 801012, Komet Brasseler, Lemgo, Germany). Then, the incisal edge was reduced by 1.5 to 2 mm in relation to the planned final length of the porcelain veneer (based on the index II and the vertical grooves). The leveling of the labial surface was performed in three different planes: the cervical, the middle, and the incisal. The preparation on the incisal edge was finished with a butt joint. In the case of a sound proximal tooth structure, no interproximal preparation was conducted. Otherwise, in the case of existing composite restorations on the interproximal area, a “wrap around” veneer was performed. Next, the retractive cord #000 (Ultradent, Indaiatuba, Brazil) (without hemostatic agent) was delicately placed using a dental explorer (DG 16 mg 6, HU FRIEDY, USA) into the gingival sulcus for a minimal gingival retraction. The margin of the preparation was brought closer to the gingiva and the outline was clearly marked (bur no. 6844014, Komet Brasseler, Lemgo, Germany). Finally, the surface was smoothed with a silicon polisher no. 9608 (Brownie Point, Komet Dental, Lemgo, Germany) on a contra-angle handpiece with copious water cooling (speed of 5000 rpm). After polishing, all clearly visible imperfections such as sharp edges and unrounded angles were corrected with a gentle motion of the red ring contra-angle handpiece (Synea, WK-99L; W&H, Austria) and fine diamond bur (no. 8868 314 016, Komet Dental, Lemgo, Germany).

### 2.4. Impression Procedure and Occlusion Registration

Next, a second retraction cord was soaked with a hemostatic agent (cord #0, Ultradent, Indaiatuba, Brazil) and gently placed in the gingival sulcus as described above, and left for 5 to 10 min. Just before the impression, the second retraction cord was removed from the gingival sulcus and the medium body silicon material (Variotime Medium Flow, Heraeus Kulzer, Hanau, Germany) was syringed directly from an Automix system along the gingival margin and on all surfaces of the prepared teeth. The metal impression tray selected based on the width of the dental arch was filled with Automix Heavy Tray a-silicon (Variotime Tray, Heraeus Kulzer GmbH, Germany) and placed on the dental arch and stabilized. 

Occlusion was registered with Aluwax bite wax in the maximum intercuspation position (MIP). The impression of the opposing arch was made with alginate impression material and immediately poured with stone. 

### 2.5. Temporalization 

The temporary veneers were obtained with the temporary resin (Protemp, 3MESPE, USA) using previously fabricated silicon index I. Provisional veneers remained seated on the teeth thanks to material retention in the interproximal areas; part of the material was left in this area for adequate maintenance until the next visit. Any excess of the material around the gingival papilla was meticulously removed using scalpel no.12 or an Excesso instrument (LM Dental, Turku, Finland). The remaining excess was gently removed with a bur no. 889540009 to avoid bleeding. 

### 2.6. Laboratory Procedure

All porcelain veneers were fabricated in the dental laboratory (by a skilled dental technician) by means of a traditional approach used for the feldspathic porcelain. 

### 2.7. Clinical Try-In and Luting Procedure 

The temporary veneers were carefully removed with a solid curette. Next, the veneers were positioned on the teeth and the fit was examined. The interproximal contacts and color were evaluated.

The teeth were isolated with a rubber dam (Nic Tone Dental Dam, thick, mint, MDC dental, Zapopan, Mexico) using the Hygienic B5, B6, or Brinker clamps (Hygienic, Coltene Whaledent, Germany) and the porcelain veneers were again tried in to check for any interferences with the clamps. 

The porcelain veneers were cleaned with 70% alcohol and etched with 9% hydrofluoric acid (Ultradent™ Porcelain Etch, USA) for 90 s. Next, the hydrofluoric acid was rinsed from the inner surface of the porcelain veneer with a water spray for 30 s, placed for 5 min in an ultrasound bath, air-dried and silanized with a minimum of three layers of silane (Ultradent Products, South Jordan, UT, USA) for 60 s. Then, the adhesive system EnaBond Seal (Micerium, Genova, Italy) was applied as one layer spread on the inner surface of the porcelain veneer, very thoroughly blown with air-spray to the thin layer, and was protected from strong sources of light to avoid the accidental activation of polymerization.

The prepared tooth was sandblasted with an abrasive unit (27 μm aluminum oxide powder; 40 PSI) in order to remove contaminations such as blood, dental plaque, or materials used for the provisional veneers. Then, orthophosphoric acid (Conditioner36, Dentsply DeTrey, Gmbh, Konstanz, Germany) with the consistency of a gel was applied over the entire preparation surface and actively spread for 20 s and meticulously rinsed with water for 10–20 s and dried. The adhesive system (Ena Bond, Micerium, Genova, Italy) was applied precisely to the entire preparation surface by rubbing in successive layers, and then very thoroughly blown to the thin and homogenous layer before polymerization. The adhesive system was polymerized for 20 s using an LED curing unit (Elipar, 3M ESPE St Paul, MN, USA). Conventional resin composite Enamel Plus (Micerium, Genova, Italy) material (UD3) was heated in a composite heating conditioner (ENA Heat, Micerium, Genova, Italy) up to 55 °C. Next, a thin layer of the heated composite was applied on the entire inner surface of the porcelain veneer. The porcelain veneers were placed on the corresponding teeth and pressed with fingers to reach the desired position (the porcelain veneer–tooth margin was meticulosity inspected). The excess of luting composite was removed from the buccal and palatal surface using a dental probe wetted in unfilled resin (ENA Seal Bond, Micerium, Genova, Italy). On the interproximal surfaces, the excess material was removed with dental floss (Oral B Satin floss, Procter & Gamble, Cincinnati, OH, USA). Next, the porcelain veneers were polymerized for 3 s on the labial surface and the procedure of removing the excess material was repeated. All of the margins were covered with glycerin gel and the final polymerization was carried out for 60 s on each surface (labial side gingivally, the incisal edge, and palatal surface close to the incisal edge). After the final polymerization, the excess composite resin was removed with scalpel no. 12 (Swann Morton, Sheffield, England). 

### 2.8. Occlusal Adjustment and Polishing 

Any premature contacts were removed from the porcelain veneer. The patient was seated to assure the upper body position at the inclination of 45°. Then, the fingertips of the operator were placed on the teeth with the porcelain veneers and the patient was asked to bite repeatedly in the MIP. If finger vibrations (fremitus) were detected, the dentist corrected the premature contacts immediately with a diamond bur Komet # 368-016 Bud FG coupled to a W&H contra-angle. A 200 μm horseshoe articulating paper was used to detect premature contacts in order to provide a “base” for the thin and more accurate 16 μm red articulation foil. The premature contact points marked on the front teeth with red and blue articulating paper were eliminated. Then, the patient was seated in the upright position (90°), a 200 μm horseshoe articulating paper was positioned between the teeth, and the patient was asked to simulate chewing a piece of gum. This test was supposed to mimic real chewing while eating [24]. Any extensive blue surfaces (representing an overload between the maxillary and mandibular anterior teeth) that appeared on the front teeth, especially on the palatal surfaces of the porcelain veneers were eliminated. Restoration margins were polished with silicone polishers (Astropol FP, HP, Ivoclar Vivadent, Schaan, Liechtenstein) and interproximal polishing strips (Soft-Lex Finishing Strips, 3M ESPE, Seefeld, Germany).

All patients received written hygiene recommendations in order to avoid using hard toothbrushes or abrasive toothpastes.

### 2.9. Evaluation Procedures

An independent and blinded calibrated operator performed follow-up examinations. The patients were examined clinically and with intraoral periapical X-ray. The clinical evaluation included: secondary caries, marginal adaptation, marginal discoloration, color match, restoration integrity, and surface roughness according to modified United States Public Health Service (USPHS) criteria (Table 2).

**Figure 1:**
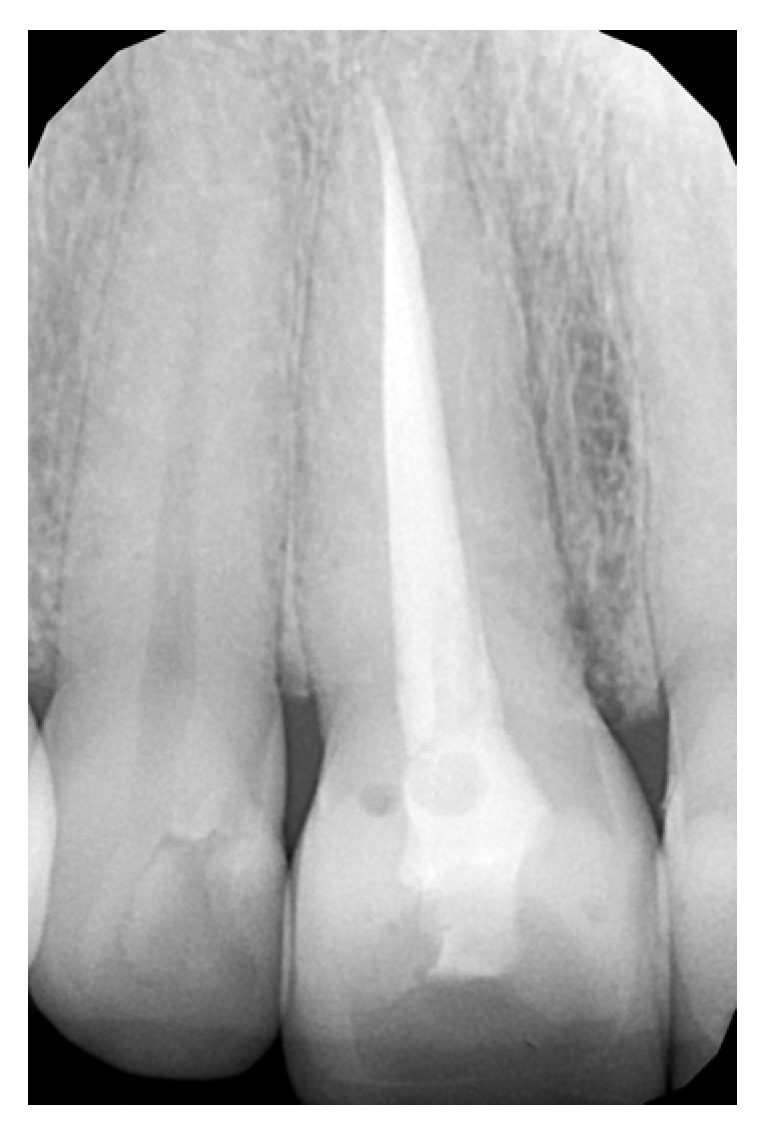
RVG image showing the endo-treated teeth.

**Figure 2:**
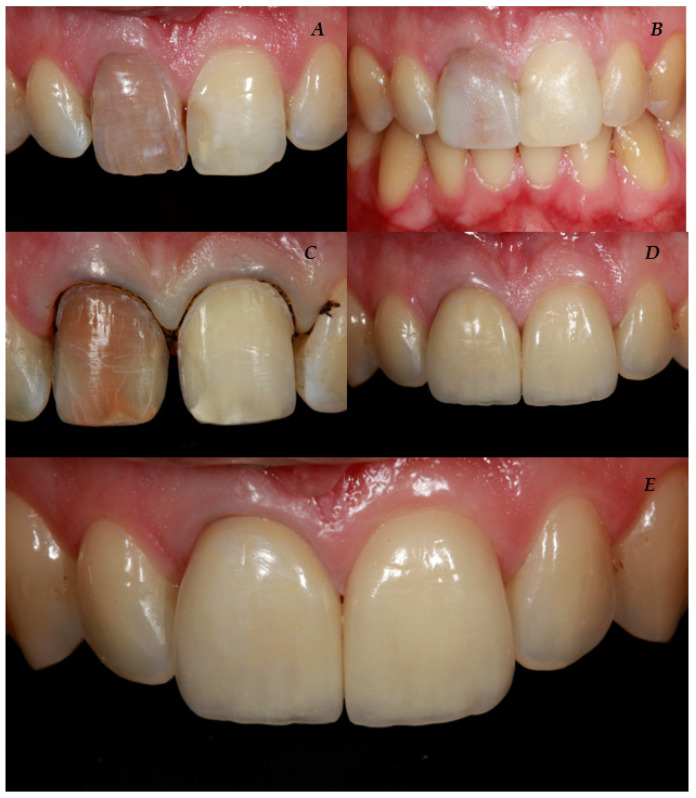
Description of the veneer preparation of two upper central incisors. (**A**) Preoperative situation; (**B**) mock-up; (**C**) tooth preparation procedure; (**D**) immediately after cementation; and (**E**) 6 years follow-up.

Figure 1 and Figure 2 show a case of the veneer preparation of two upper incisors.

### 2.10. Statistical Analysis

The performance of the restorations was assessed using the Mann–Whitney non-parametric statistical analysis. The level of significance was set at *p* < 0.05. The statistical analyses were conducted using the SigmaPlot software (SigmaPlot 12.0, SPSS Inc., Chicago, IL, USA).

## 3. Results

The qualitative evaluation using USPHS criteria for the restorations evaluated is shown in Table 3. All restorations were considered acceptable; however, one porcelain veneer cemented on a VT failed due to secondary caries. Despite this, there were no statistically significant differences in any of the criteria evaluated (*p* ≤ 0.671). 

## 4. Discussion

A longitudinal study was conducted evaluating the clinical behavior of indirect porcelain veneers performed in VT and NVT. USPHS Evaluation System criteria were used, as suggested in the literature [25,26,27]. The qualitative evaluation showed acceptable results for all of the restorations evaluated, although one porcelain veneer cemented on a VT failed due to secondary caries. All in all, the survival rate of these restorations and all of the characteristics were satisfactory after 8-year clinical performance. 

Previous longitudinal clinical reports have assessed the performance of dental ceramic veneer restorations and have proven good clinical performance, outstanding aesthetics, and patient fulfilment [16,18,19,21]. In the present study, the porcelain veneers exhibited a higher survival rate of 97.9%–100% after 8 years of performance, which is supported by others reporting survival rate of 91% to 100% [28,29]. A survival rate varying from 80.1 to 100% was found after a follow-up of less than 5 years [30] and of 47 to 100% after 5 to 7 years of clinical service [31,32,33]. In addition, studies with a follow-up of 10 to 12 years presented a survival rate of 53 to 94.4% [22,29,34].

Indirect veneers can be used as an alternative to full-coverage restorations, since they prevent the aggressive preparation and removal of the palatal tooth structure, therefore preserving dental structure [35]. However, there are many possible well-known failures that can occur to the ceramic veneers including debonding, chipping, fracture, margin discoloration, or secondary caries. Secondary caries was also known as a lesion at the margin level of an existing restoration [33]. In this study, this was only found in the case of one restoration. This particular porcelain veneer was cemented onto the tooth with both mesial and distal composite restorations (class IIIMD). This particular case belongs to a female patient who went through a stressful period in her life and did not perform her mouth care in a proper manner. Both factors—the stress, which can be a cause of xerostomia, and the improper hygiene—could be the reasons for the secondary caries [36]. 

In addition, the presence of secondary caries at the veneer–tooth interface could be explained by various other factors. Poor oral hygiene, caries susceptibility, and saliva or blood contamination due to the lack of rubber dam isolation during the cementation procedure were described as possible reasons [37]. In this retrospective study, the rubber dam was placed in all cases and the patients were monitored by means of professional hygiene. It was shown that the restorations performed under rubber dam isolation developed a lower failure rate than restorations completed with saliva ejectors and cotton rolls only [37]. In summary, patient- and operator-related factors influence the success rate. Low-risk patients under controlled settings might be a reason for the lower level of secondary caries lesions [38,39,40]. This supports the findings of this study, as secondary caries lesions developed in only one case.

It is worth emphasizing that, in terms of the clinical success of ceramic restorations, a marginal fit was considered an important factor [41,42,43]. The external marginal adaptation of ceramic veneers, which is expressed as the vertical distance between the margins of the fabricated veneers and the finish line of the prepared tooth, significantly influences the success rate of the restoration [43]. Since this parameter was acceptable both for VT and NVT, the restorations exhibited good marginal adaptation, therefore minimizing the contact surface of the cement with the oral environment [41]. On the other hand, the internal marginal adaptation is defined by a measurement of the cement film thickness under the dental restoration and is notably prejudiced by the accuracy of the fabrication procedure used [44]. In case of poor internal marginal adaptation, a negative correlation can occur between the thickness of the luting cement and the stress distribution on the inner and outer surfaces of the veneer, which could lead to crack propagation within the restoration [45]. It seems that all of the porcelain veneer restorations were accurate in terms of the fabrication process, which could support the finding of the present study.

For the restoration integrity, the combination of minimal preparation through mock-up provided the maximal conservative approach [46]. A butt joint design was used in this study, as suggested by Castelnuovo et al., who proved the best performance of this preparation modality [47]. It provides the enhanced bonding between the tooth structure and the ceramic material as a consequence of keeping the peripheral enamel layer around the margins and preventing microleakage formation, especially at the interface on the palatal surface, owing to an improved shear stress distribution [47]. Moreover, the literature revealed that significantly better marginal adaptation is observed when etch-and-rinse method along with primer and adhesive were applied [48].

The differences in the marginal discoloration between VT and NVT were not statistically significant in this study. A previous retrospective study with a follow-up of 10 years yielded a survival rate of 93.5%, and 82.8% after 20 years [49]. Beier et al. [49] considered the marginal discoloration as a minor complication since it occurred in 21.3% of cases, predominantly in smokers. This observation is not supported by the present study, where no discoloration with deep penetration of the restoration margins was perceived, and the survival rate was higher. However, both studies cannot be compared due to the different restoration geometry and diverse inclusion and exclusion criteria.

Additionally, all restorations exhibited satisfactory stable color behavior. The color of the veneer restoration matched the color of the VT and endodontically treated teeth. It is important to emphasize that due to the close cooperation between the laboratory and the dentist, the color of the restoration was evaluated individually. The shade, thickness, and type of ceramic materials affected the final color of the ceramic veneer restorations since a target color of the veneer and tooth cannot often be chosen by the practitioner [50,51,52]. Diverse resin cement shades could be selected to modify the final color outcome of the ceramic veneer restoration [50,51,52]. In all cases, the same shade was applied, thus no difference in color match was observed. In addition, the thickness of a ceramic veneer restoration is restricted by the minimal amount of tooth preparation and target restorative space.

One should bear in mind that the color of endodontically treated teeth is frequently compromised [53]. Diverse restorative approaches can be considered for discolored teeth starting from NVT bleaching, direct composite restorations, direct composite veneers, indirect veneers, and ending with ceramic crowns [54]. In the past, more invasive treatment alternatives such as crowns were frequently applied; however, recently, more conservative options are preferred in order to predictably restore the endodontically treated anterior tooth. However, there are still controversies among practitioners as to whether a porcelain veneer on a NVT is a reliable option and whether it can be widely recommended [55].

The composition and the surface structure of a dental restorative material impacts the initial bacterial adhesion. A rough material surface will promote more plaque formation [56]. During the evaluations with the follow-up approaches of the restorations presented in this study, no signs of porosity, defect, scratching, or disintegration on the surface were observed. These outcomes might be the reason for the highly polished feldspathic porcelain material used in this study both for treating VT or NVT [57,58]. Additionally, the patients were educated by a dental hygienist to avoid brushing with hard toothbrushes or abrasive toothpaste.

Some limitations could be found in the present study, including that only one adhesive technique, using the same adhesive system and resin cement, was used. The other limitations represent the relatively low number of restorations, the need for multi-center studies, the presence of pre-existing composite restorations, adhesive surface, the inhomogeneous status of the teeth within the study group, the difference between subjects regarding the occlusal relationship, and relatively different occlusal conditions that are difficult to calibrate ideally in the clinical studies. Moreover, different types of ceramics could be evaluated in further research including computer-aided design–computer-aided manufacturing (CAD-CAM)-based materials. Finally, a longer follow-up period is necessary to establish more conclusive findings.

## 5. Conclusions

Within the limitations of the current study, it can be concluded that the ceramic veneers showed a satisfactory clinical performance both on VT and NVT.

## Figures and Tables

**Table 1:** Distribution of porcelain veneer restorations.

Independent Variable	n	%
Sex		
Male	13	23.6
Female	42	76.3
**Total**	55	100
**Tooth type**		
Central incisor	81	52.6
Lateral incisor	53	34.4
Canine	20	13.0
**Total**	154	100
**Follow-up time (years)**		
0.5–2	3	1.9
2–3.9	1	0.7
4–5.9	43	27.9
6–7.9	22	14.3
More than 8	85	55.2
**Total**	154	100
**Tooth vitality**		
Vital	93	60.4
Non-vital	61	39.6
**Total**	154	100

**Table 2:** Modified United States Public Health Service criteria used for restoration assessment.

Category	Criterion	Definition
Secondary Caries	ALPHA	No evidence of caries
	CHARLIE	Caries is evident, contiguous with the margin of the restoration
Marginal Adaptation	ALPHA	Restoration is contiguous with existing anatomical form, explorer does not catch
	BRAVO	Explorer catches, no crevice is visible into which explorer will penetrate
	CHARLIE	Obvious crevice at margin, dentine or base exposed
	DELTA	Restoration mobile, fractured partially or totally
Marginal Discoloration	ALPHA	No discoloration evident
	BRAVO	Slight staining: can be polished away
	CHARLIE	Obvious staining: cannot be polished away
	DELTA	Gross staining
Color Match	ALPHA	Very good color match
	BRAVO	Slight mismatch in color, shade, or translucency
	CHARLIE	Obvious mismatch, outside the normal range
	DELTA	Gross mismatch
Restoration Integrity	ALPHA	No material defect, no crack
	BRAVO	Two or more cracks not compromising marginal integrity or contacts
	CHARLIE	Restorative fractures compromising marginal integrity or contacts
	DELTA	Partial or complete restorative loss
Surface Roughness	ALPHA	Smooth surface
	BRAVO	Slightly rough or pitted
	CHARLIE	Rough, cannot be refinished
	DELTA	Surface deeply pitted, irregular grooves

**Table 3:** Clinical evaluation of anterior porcelain veneers: comparison between vital and non-vital teeth, according to the United States Public Health Service criteria.

	Vital Teeth		Non-Vital Teeth		Mann–Whitney *p*
	Restoration Scores (A/B/C/D)	Restorations Clinically Acceptable	Restoration Scores (A/B/C/D)	Restorations Clinically Acceptable	
Secondary Caries	92/1/-/-	98.9%	61/0/-/-	100%	0.188
Marginal Adaptation	66/27/0/0	100%	52/9/0/0	100%	0.635
Marginal Discoloration	66/27/0/0	100%	46/15/0/0	100%	0.871
Color Match	93/0/0/0	100%	59/2/0/0	100%	0.648
Restoration Integrity	91/2/0/0	100%	60/1/0/0	100%	0.867
Surface Roughness	92/1/0/0	100%	60/1/0/0	100%	0.893

## Data Availability

Not applicable.
